# Developmental and conditional regulation of DAF-2/INSR ubiquitination in *Caenorhabditis elegans*

**DOI:** 10.1093/g3journal/jkaf009

**Published:** 2025-01-22

**Authors:** Ivan B Falsztyn, Seth M Taylor, L Ryan Baugh

**Affiliations:** Department of Biology, Duke University, Durham, NC 27708, USA; Department of Biology, Duke University, Durham, NC 27708, USA; Department of Biology, Duke University, Durham, NC 27708, USA

**Keywords:** *C. elegans*, DAF-2, insulin, IGF, ubiquitin, CHIP, starvation, L1 arrest

## Abstract

Insulin/IGF signaling (IIS) regulates developmental and metabolic plasticity. Conditional regulation of insulin-like peptide expression and secretion promotes different phenotypes in different environments. However, IIS can also be regulated by other, less understood mechanisms. For example, stability of the only known insulin/IGF receptor in *Caenorhabditis elegans*, DAF-2/INSR, is regulated by CHIP-dependent ubiquitination. Disruption of *chn-1/CHIP* reduces longevity in *C. elegans* by increasing DAF-2/INSR abundance and IIS activity in adults. Likewise, mutation of a ubiquitination site causes *daf-2(gk390525)* to display gain-of-function phenotypes in adults. However, we show that this allele displays loss-of-function phenotypes in larvae and that its effect on IIS activity transitions from negative to positive during development. In contrast, the allele acts like a gain-of-function in larvae cultured at high temperature, inhibiting temperature-dependent dauer formation. Disruption of *chn-1/CHIP* causes an increase in IIS activity in starved L1 larvae, unlike *daf-2(gk390525)*. CHN-1/CHIP ubiquitinates DAF-2/INSR at multiple sites. These results suggest that the sites that are functionally relevant to negative regulation of IIS vary in larvae and adults, at different temperatures, and in nutrient-dependent fashion, revealing additional layers of IIS regulation.

## Introduction

Insulin/IGF signaling (IIS) regulates growth, development, metabolism, stress resistance, and aging in metazoans. In the nematode *Caenorhabditis elegans*, the sole known insulin/IGF receptor is encoded by *daf-2/INSR* (Murphy and Hu 2013). DAF-2/INSR signals through a conserved phosphoinositide 3-kinase (PI3K) pathway including AGE-1/PI3K, PDK-1, AKT-1, and AKT-2 to govern nuclear localization and activity of the transcription factor DAF-16/FOXO.

When worms hatch in the absence of food, they remain in a developmentally arrested state in the first larval stage known as L1 arrest (or L1 diapause) (Baugh 2013). Extended starvation during L1 arrest causes developmental abnormalities of the gonad, including germline tumors (Jordan *et al.* 2019). Worms also arrest development as dauer larvae in the third larval stage in response to adverse environmental conditions including high population density, limited nutrient availability, and high temperature (Hu 2007). IIS regulates L1 arrest and dauer formation, with *daf-2/INSR* mutants displaying constitutive arrest phenotypes as L1 and dauer larvae (Gems *et al.* 1998; Baugh and Sternberg 2006) as well as increased starvation resistance, including increased survival of L1 arrest (Muñoz and Riddle 2003; Baugh and Sternberg 2006) and suppression of starvation-induced developmental abnormalities (Jordan *et al.* 2019). IIS also regulates adult physiology, and disruption of *daf-2/INSR* substantially increases lifespan and suppresses vitellogenesis (Cynthia Kenyon *et al.* 1993; Murphy *et al.* 2003; Depina *et al.* 2011).

Ubiquitin is a small peptide that is covalently attached to proteins. Ubiquitination often targets proteins for degradation, but it can regulate protein function in other ways as well (Kipreos 2005; Zheng and Shabek 2017). The quality control E3 ubiquitin ligase CHIP mono-ubiquitinates worm, fly, and human INSR. The worm ortholog DAF-2/INSR is ubiquitinated at multiple sites (Tawo *et al.* 2017). DAF-2/INSR stability is regulated by the sole worm ortholog of CHIP, encoded by gene *chn-1*. Disruption of *chn-1/CHIP* increases DAF-2/INSR abundance in adults, increasing IIS activity and reducing lifespan (Tawo *et al.* 2017). *daf-2(gk390525)* is a point mutation resulting in a K1614E amino acid substitution, affecting one of the lysine residues targeted by CHN-1/CHIP-dependent ubiquitination (Tawo *et al.* 2017). This mutant has reduced lifespan (Tawo *et al.* 2017; Zhao *et al.* 2021) and increased vitellogenesis (Kern *et al.* 2021), consistent with increased IIS activity, causing it to be described as a gain-of-function allele. Although the effects of *chn-1/CHIP* and DAF-2/INSR ubiquitination have been documented in adults, it is unknown whether or how ubiquitination affects IIS during larval development or in conditions where there are large differences in IIS, such as in fed vs starved animals.

We characterized *daf-2(gk390525)* and *chn-1(by155)* phenotypes in larvae, during L1 arrest and recovery as well as dauer formation, in addition to validating previously described adult phenotypes. We complemented phenotypic analysis with quantification of DAF-16/FOXO subcellular localization as a proxy for IIS activity. We confirm published results showing that this allele causes increased IIS activity in adults. We also show that the allele increases IIS activity during temperature-dependent dauer formation. However, we demonstrate that *daf-2(gk3902525)* reduces IIS activity during larval development and L1 arrest, behaving like a loss-of-function allele with a dominant-negative effect. In contrast to *daf-2(gk390525)*, disruption of *chn-1/CHIP* increases IIS during L1 arrest, suggesting regulation of DAF-2/INSR through ubiquitination of 1 or more amino acid residues other than K1614. These results demonstrate that *chn-1/CHIP* and ubiquitin-dependent regulation of DAF-2/INSR varies during development and in different conditions. They also highlight that *daf-2(gk390525)* has complex effects on IIS, warranting caution in interpretation of phenotypic analysis.

## Materials and methods

### Worm maintenance

All worms were maintained at 20°C on nematode growth medium (NGM) plates seeded with *E. coli*  OP50. Worms were well-fed for at least 5 generations before experimental use. With the exception of scoring starvation-induced gonad abnormalities, experiments were carried out on NGMs seeded with OP50. All experiments were conducted at 20°C unless otherwise noted. WormBase was used for general queries regarding genes and strains (Sternberg *et al.* 2024). All strains obtained for this work (Table 1) were backcrossed at least 4 times unless at least 4 generations of backcrossing were previously noted. Fluorescence microscopy was used for identifying fluorescent strains in all reporter lines generated, and PCR and gel electrophoresis were used for genotyping all mutants generated in this work (Table 2).

**Table 1.** Published strains.

Strain	Genotype	Description	Source
N2	Wild type		
CB1370	*daf-2(e1370)*	Substitution: P1465S, CCA to TCA (Kimura *et al.* 1997)	CGC
IC166	*daf-18(ok480)*	Deletion: 956 bp (*C. elegans* Deletion Mutant Consortium 2012)	Ian Chin-Sang—Queen's University
GA1990	*daf-2(gk390525)*	Substitution: K1614E, AAA to GAA (Tawo *et al.* 2017)	David Gems—University College London
BR2823	*chn-1(by155)*	Deletion: 989 bp (Hoppe *et al.* 2004)	CGC
RT130	*pwIs23* [VIT-2::GFP]	Multicopy transgene (Balklava *et al.* 2007)	CGC
OH16024	*daf-16*(*ot971*[DAF-16::GFP])	Endogenous fusion (Aghayeva *et al.* 2020)	Oliver Hobert—Columbia University
LRB455	*daf-16(ot971*[DAF-16::GFP]); *daf-18(ok480)*	Cross: IC166 × OH16024 (Chen *et al.* 2022)	Ryan Baugh—Duke University
PD4667	*ayIs7* [*hlh-8p::gfp* + *dpy-20*(+)]	*hlh-8* transcriptional reporter for M cell identification (Harfe *et al.* 1998)	CGC
LRB477	*daf-18(ok480)*; *ayIs6*[*hlh-8p*::GFP + *dpy-20*(+)]	Cross IC166 × PD4667 (Chen *et al* 2022)	Ryan Baugh—Duke University

**Table 2.** Generated strains.

Strain	Genotype	Description	Source
LRB537	*pwIs23* [VIT-2::GFP]; *daf-2(e1370)*	Cross: RT130 × CB1370	This work
LRB607	*pwIs23* [VIT-2::GFP]; *daf-2(gk390525)*	Cross: RT130 × GA1990	This work
LRB562	*daf-16*(*ot971*[DAF-16::GFP]); *daf-2(gk390525)*	Cross: OH16024 × GA1990	This work
LRB602	*daf-16*(*ot971*[DAF-16::GFP]); *daf-2(e1370)*	Cross: OH16024 × CB1370	This work
LRB608	*daf-16*(*ot971*[DAF-16::GFP]); *chn-1(by155)*	Cross: Oh16024 × BR2823	This work
LRB441	*chn-1(by155)*; *daf-2(e1370)*	Cross: BR2823 × CB1370	This work
LRB442	*chn-1(by155)*; *daf-2(gk390525)*	Cross: BR2823 × GA1990	This work
LRB664	*daf-2(gk390525)*; *ayIs6*[*hlh-8p*::GFP + *dpy-20(+)*]	Cross: BR2823 × PD4667	This work
LRB665	*chn-1(by155)*; *ayIs6*[*hlh-8p*::GFP + *dpy-20(+*)]	Cross: GA1990 × PD4667	This work

### Lifespan

Seven L4 larvae were picked and allowed to lay eggs overnight (∼16 h) before being removed to obtain a synchronized population. Twenty-five progeny in the L4 stage were picked per 10-cm plate for 2 total plates per biological replicate for each strain used. Day 1 was defined as the first day of adulthood. Worms were transferred to fresh plates daily throughout egg laying. Worms were determined to be dead if not moving and/or unresponsive to gentle prodding with a transfer pick every 24 h. Worms that crawled off the plate, died, burrowed, or otherwise were not confirmed as dead were censored. Kaplan–Meier estimations, log-rank tests, and other statistics were calculated using the online OASIS 2 application (Han *et al.* 2016). *daf-2(gk390525)* was backcrossed to the laboratory N2 4 times before analyzing lifespan.

### Fluorescence imaging of *vit-2* reporter

A synchronized population of worms was obtained through a timed egg lay, by picking 7 L4 larvae and allowing them to lay eggs overnight (∼16 h) and then removing them. Progeny were grown to early adulthood during which a single row of embryos was observed in the gonad and a few unhatched embryos were seen on the lawn. Approximately 20–40 animals were picked into 10 mM levamisole on 4% noble agar pads. Worms were imaged at a total magnification of 100× using an AxioImager compound microscope (Zeiss) and an AxioCam 506 Mono camera. Fiji was used for basic image processing, including rotating, cropping, file conversion, etc.

### L1 starvation culture preparation

Seven L4 larvae were picked onto 10-cm NGM plates seeded with OP50. After 96 h at 20°C, plates were washed with S-basal medium and hypochlorite treated to obtain embryos (Stiernagle 2006). However, for starvation cultures used for gonad abnormality scoring, bleach plates were prepared by picking 8 early adults (just after the onset of egg laying) and were cultured for 72 h prior to hypochlorite treatment. Embryos were transferred to virgin S-basal (lacking ethanol and cholesterol) at a density of 1 embryo per microliter of media and maintained at 20°C on a tissue culture roller drum. *daf-2(gk390525)* and *chn-1(by155)* were backcrossed to the laboratory N2 4 times before analyzing starvation survival.

### Whole-animal VIT-2::GFP quantification

Embryos were obtained by hypochlorite treatment as described and allowed to hatch and arrest overnight (∼16 h) for synchronization. Progeny were grown to early adulthood during which a single row of embryos was observed in the gonad and a few unhatched embryos were seen on the lawn. Worms were washed from the plate with S-basal, anesthetized with 50 µM sodium azide, and transferred to a 96-well plate. Wells were imaged using an ImageXpress Nano automated imager at 100× total magnification. Images for the same well were stitched together, and objects were identified and filtered by size (5 µm < width < 40 µm, 20,000 pixels < area < 75,000 pixels). GFP intensity was measured by subtracting background intensity for each well from the average intensity of each individual object. Each individual object was also manually screened to exclude any incidences of multiple worms, partial worms, or extraneous debris detected as an object. The mean average intensity for each condition within each replicate was calculated, and a 2-tailed unpaired t-test was used for pairwise comparisons between genotypes.

### L1 starvation survival

Survival during L1 arrest was scored by plating a 100-μL aliquot from a starvation culture on a seeded plate just off the lawn. The number of animals plated was scored by counting the number of L1 larvae in the 100-μL aliquot. After 48 h, the number of animals that survived was scored by counting the number of live worms on the plate. The frequency of animals that survived was calculated by dividing the number of animals that survived by the number of animals plated. Survival was scored daily starting from day 1, 24 h after preparation of the starvation culture (hypochlorite treatment). Statistical comparisons were made using quasi-binomial logistic regression with the proportion of live worms as the response variable and the duration of L1 arrest as the explanatory variable. Regression was used to estimate half-lives, which were subjected to a 2-tailed unpaired t-test to compare genotypes.

### M cell divisions

M cells were identified with a GFP reporter (strain PD4667 ayIs7 [*hlh-8p::gfp*]). Arrested L1 larvae were prepared as described above. Three or eight days after hypochlorite treatment (see figure legend), they were mounted on 4% agarose pads and viewed at 400× total magnification on a Zeiss AxioImager compound microscope, and the number of M cells was scored in each of ∼100 larvae.

### Growth rate

Five hundred L1 larvae starved in L1 arrest for 8 days or 1 day (actually ∼12 h, given ∼12 h to complete embryogenesis after hypochlorite treatment) as a control were plated and cultured for 48 h at 20°C. Worms were then washed with S-basal and transferred to clean unseeded NGM plates. 50–100 worms were imaged using a Zeiss SteREO Discovery.V20 stereo microscope and an AxioCam MrM camera at 20× total magnification for worms starved for 1 day and 30× total magnification for worms starved for 8 days. The Fiji plugin WormSizer was used to measure worm length (Moore *et al*. 2013). A linear mixed-effects (lme) model was fit using body length as the response variable, the interaction between starvation condition and genotype as the fixed effects, and experimental replicate as the random effect (R example: lme(length∼strain × day), random = ∼1|replicate, data = wormsizer).

### Brood size

Approximately 50 L1 larvae starved in L1 arrest for 8 days or 1 day (actually ∼12 h, given ∼12 h to complete embryogenesis after hypochlorite treatment) as a control were plated and allowed to grow for 48 h at 20°C. Eighteen larvae were then singled onto fresh 6-cm plates. Worms were transferred to fresh plates daily, and the number of progeny was counted after 48 h until the number of progeny laid in a day reached 0. Worms that arrested after singling, crawled off the plate, died, burrowed, or otherwise were not able to lay a complete brood for reasons other than sterility were censored. Total brood size was calculated by adding all days of egg laying. A linear mixed-effect model was fit to the data as described for growth rate, however, using total brood size as the response variable.

### RNA interference

*Escherichia coli*  HT115 was used for RNAi by feeding. The *chn-1* RNAi bacterial strain was obtained from the Ahringer RNAi library. The *daf-2* RNAi bacterial strain was obtained from the Cynthia Kenyon Lab (Dillin *et al.* 2002). Empty vector RNAi bacteria carried the L4440 plasmid. Frozen stocks were streaked onto Luria–Bertani (LB) plates with carbenicillin (carb) (100 mg/mL) and tetracycline (tet) (12.5 mg/mL), and single colonies were transferred to 1 mL of LB with carb and tet at the same concentrations. After 16 h of incubation at 37°C while shaking, 100 μL of culture was transferred to 5 mL of Terrific broth with carb (50 mg/mL) and incubated for 16 h at 37°C while shaking. After incubation, cultures were centrifuged at 4000 rpm for 10 min and resuspended in S-complete medium with 15% glycerol and aliquoted for freezing. 15 μL from single-use frozen stocks was used to seed lawns on NGM with carb and IPTG plates which were spread to cover ∼60% of the plate surface and allowed to grow at room temperature overnight.

### Gonad abnormalities

Approximately 150 arrested L1 larvae were plated onto NGM + Carb + IPTG plates seeded with *E. coli*  HT115 carrying the indicated RNAi plasmid or the L4440 plasmid as an empty vector control. HT115 carrying L4440 was used as food in all experiments where gonad abnormalities were assayed even when RNAi was not part of the experimental design. Worms were cultured until early adulthood, which varied by genotype. *daf-2(e1370)* mutants are slow growing, and recovered worms were cultured for 96 h. N2 worms developed at approximately the same rate across RNAi treatments and were scored ∼72 h after plating L1s. Both *daf-2(gk390525)* and *chn-1(by155)* developed at about the same rate as N2 and were also scored after ∼72 h. Worms were then washed from plates with S-basal including 10 mM levamisole and transferred to 4% noble agar pads on a microscope slide. Worms were viewed at 200× total magnification using Nomarski microscopy on a Zeiss AxioImager compound microscope. Worms with proximal germ cell tumors or uterine masses, as described in Jordan *et al.* (2019), were classified as abnormal. Worms with other, relatively rare abnormalities were censored, unlike Jordan *et al.* (2019). Approximately 50 animals were scored per condition, and abnormality frequency was calculated by dividing the number of abnormal worms by the total number of worms scored. Bartlett's test was used to test homogeneity of variance across replicates and conditions. If variance was not found to be different across these groups (*P* > 0.05), then 2-tailed, unpaired, pooled variance t-tests were used to compare the frequency of abnormalities across pairs of conditions or genotypes. If Bartlett's test indicated that variance significantly differed across groups, then the same t-tests were performed except variance was not pooled across groups. A 2-way ANOVA was used for 2-factor comparisons in the case of double mutants, combining mutants and RNAi, and double RNAi treatments, and the *P*-value for the interaction between factors was used to assess epistasis.

### Scoring abnormalities in heterozygotes

Starvation cultures with heterozygotes were prepared by hypochlorite-treating gravid wild-type hermaphorites mated with mutant males. 24 h before hypochlorite treatment, 50 wild-type hermaphrodite L4 larvae and 50 male L4 larvae carrying the single-copy DAF-16::GFP knock-in allele *daf-16(ot971[daf-16::GFP])* and indicated mutation were picked onto a 6-cm NGM plate with food. Smaller plates were used to increase the chance of encounters between males and hermaphrodites and maximize mating efficiency. Males carrying the DAF-16::GFP knock-in allele were used so that cross-progeny could be unequivocally identified during scoring. This allele was an ideal choice as it is a single-copy knock-in that does not interfere with protein function and can be easily identified in early adults. 24 h after preparing the cross plate, starvation cultures were prepared as described. After 8 days, L1 larvae were plated onto empty vector RNAi HT115 bacteria and grown to early adulthood. Worms were transferred to slides as previously described, and heterozygotes were identified and scored by the presence of DAF-16::GFP.

### Scoring abnormalities in progeny of heterozygotes

Starvation cultures were prepared with all the progeny of heterozygous mothers (i.e. 25% homozygous, 50% heterozygous, and 25% wild type). Eight male L4 larvae carrying the indicated mutation and the single-copy DAF-16::GFP knock-in allele *daf-16(ot971[daf-16::GFP])* were crossed to a single wild-type hermaphrodite L4. After 48 h, heterozygous cross-progeny were identified by the presence of the DAF-16::GFP reporter, and 50 GFP(+) L4 hermaphrodites were transferred to a fresh plate. Wild-type or homozygous mutants scored as controls also carried the DAF-16::GFP allele, but were not crossed, though 50 L4 larvae were transferred to a fresh plate. After 24 h, gravid adults were washed and hypochlorite treated as previously described. Arrested L1s were starved for 8 days before being plated onto empty vector HT115 bacteria and grown to early adulthood. Worms were transferred to slides, and 50 individuals were scored for the presence of gonad abnormalities.

### Dauer formation

Seven well-fed L4 larvae were transferred to fresh plates and cultured for 20 h at the indicated temperature. Adults were removed, and plates were returned to the indicated growth temperatures for 48 h. Dauers were identified visually by morphology (narrow body, elongated) and increased refractive index. The number of dauers on the plate was then counted, and the frequency of dauers was calculated by dividing the number of dauers by the total population size. Statistical comparisons were made similarly to those described for gonad abnormalities. Bartlett's test was used to test for equal variances, and where variances were not unequal, pooled variance unpaired t-tests were used to compare dauer frequency between conditions. If variances were unequal, then variance was not pooled.

### DAF-16/FOXO subcellular localization

Larvae in L1 arrest were obtained as described above, and they were cultured for 18 h after hypochlorite treatment before being imaged (it takes ∼12 h to complete embryogenesis in these conditions; ∼6-h L1 arrest). Synchronized, fed early L1 larvae were obtained by hypochlorite-treating gravid worms, plating embryos directly onto food, and culturing them for 18 h (∼6-h feeding). Late L1 larvae were obtained by plating embryos directly onto food and culturing them for 24 h (∼12-h feeding). L3/L4 were cultured for 48 h (∼36-h feeding), and adults were cultured for 72 h (∼60-h feeding). Timed egg lays were used to obtain synchronized populations of L1 larvae at 20 and 27°C to mimic conditions used in the dauer formation assay—7 L4s were picked to a fresh, seeded plate and cultured for 24 h before being removed. Progeny were washed into 1.5-mL Eppendorf tubes with 1 mL of S-basal. Worms were centrifuged at 3000 rpm for 60 s and then transferred by pipetting 2 μL of volume from the pellet to a 4% noble agar pad. Slides were visualized at 1000× total magnification for early and late L1 larvae, and 400× total magnification for L3/L4s and adults, using a Zeiss AxioImager compound microscope. Subcellular localization was scored with 4 categories ranging from completely nuclear to completely cytoplasmic, as previously described (Chen *et al.* 2022). To minimize confounding environmental effects on DAF-16 localization, worms were scored for only the first 3 min after being transferred to slides. A Cochran–Mantel–Haenszel chi-squared test was used to perform pairwise comparisons between genotypes and conditions for the distribution of DAF-16/FOXO subcellular localization categories.

## Results and discussion

### *daf-2(gk390525)* behaves as a gain-of-function allele in adults

**Fig. 1.**
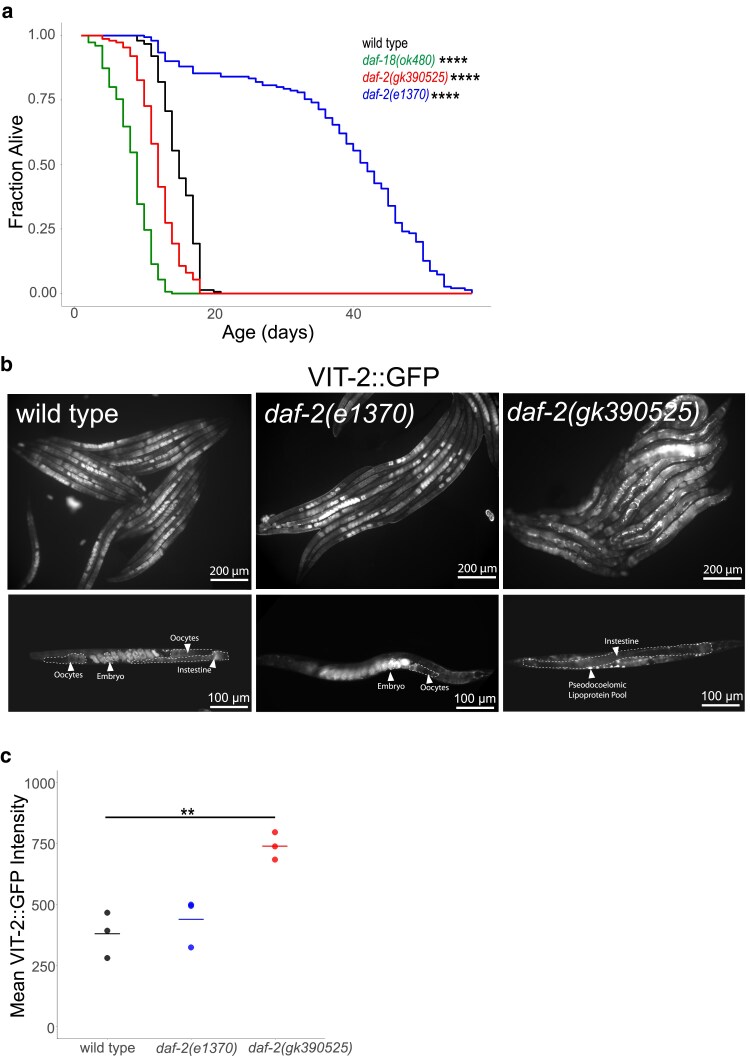
*daf-2(gk390525)* mutants demonstrate documented IIS gain-of-function phenotypes in adults. a) Adult lifespan was scored daily for 3 biological replicates with 50 worms per replicate. Replicates were pooled for presentation and statistical analysis in order to capture variation among replicates. See Supplementary Fig. 1 for individual replicates. See Supplementary Table 1 for complete results. ****P < 0.0001; log-rank test. b) Images of VIT-2::GFP expression are presented for a random population of animals (top; 200× total magnification) and representative individuals (bottom; 400× total magnification). In the representative images (bottom), dashed lines and arrowheads indicate salient features including intestine, oocytes, embryos, and pseudocoelomic lipoprotein pools. Well-fed animals were imaged during early adulthood, when a single row of embryos was visible in the uterus. To ensure matching stages, wild type and *daf-2(e1370)* were imaged after 84-h recovery from L1 arrest, and *daf-2(gk390525)* was imaged after 72 h. c) VIT-2 expression was quantified in the indicated genetic backgrounds using VIT-2::GFP intensity at 100× total magnification. Well-fed animals were imaged during early adulthood. Individual points represent the mean for a single replicate (wild type—*n* = 395, *daf-2(e1370)*—*n* = 654, *daf-2(gk390525)*—*n* = 663). Horizontal lines represent the mean intensity across 3 biological replicates within the same genotype. **P < 0.01; 2-way unpaired pooled variance t-test.

We assayed lifespan for *daf-2(gk390525)*, a reportedly gain-of-function allele (Tawo *et al.* 2017; Kern *et al.* 2021; Zhao *et al.* 2021), *daf-2(e1370)*, a class 2 loss-of-function allele (Gems *et al.* 1998), and *daf-18(ok480)*, a null allele of a negative regulator (*daf-18/PTEN*) of AGE-1/PI3K and thus IIS (Ogg and Ruvkun 1998). As expected, *daf-2(e1370)* had a significant increase in lifespan relative to the wild-type (N2) control, and *daf-18(ok480)* had a significant decrease (Fig. 1a). *daf-2(gk390525)* had a smaller, albeit significant, decrease in lifespan. These results confirm the published gain-of-function behavior of *daf-2(gk390525)* with respect to lifespan (Tawo *et al.* 2017; Zhao *et al.* 2021).

DAF-16/FOXO antagonizes vitellogenesis (Murphy *et al.* 2003; Depina *et al.* 2011), and IIS promotes vitellogenesis and yolk venting (Kern *et al.* 2021). We imaged the expression of a multicopy VIT-2 reporter gene in *daf-2(e1370)* and *daf-2(gk390525)* gravid adults on the first day of egg laying. VIT-2 and other vitellogenin proteins are synthesized in the intestine and secreted into the pseudocoelom (body cavity), and oocytes are provisioned through receptor-mediated endocytosis of vitellogenin lipoprotein particles (Perez and Lehner 2019). VIT-2::GFP expression appeared lower in the intestine and pseudocoelom of *daf-2(e1370)* compared with wild type (Fig. 1b), consistent with reduced IIS and vitellogenesis. However, embryos in utero appeared brighter in *daf-2(e1370)*, consistent with increased vitellogenin provisioning with reduced IIS (Jordan *et al.* 2019). In contrast, *daf-2(gk390525)* appeared to have a higher total signal for VIT-2::GFP, with conspicuous lipoprotein pools at increased frequency in the pseudocoelom. Such pseudocoelomic lipoprotein pools have been documented in aged wild-type adults (Ezcurra *et al.* 2018; Kern *et al.* 2021), and their precocious development is consistent with *daf-2(gk390525)* promoting vitellogenesis. We also quantified the VIT-2::GFP reporter in whole worms. Given different effects in the intestine, pseudocoelom, oocytes, and embryos, this whole-animal approach may lack sensitivity, and *daf-2(e1370)* was not significantly different from wild type. However, *daf-2(gk390525)* was significantly brighter than wild type (Fig. 1c), further supporting the conclusion that there is an overall increase in vitellogenesis. These results confirm the published gain-of-function behavior of *daf-2(gk390525)* with respect to vitellogenesis (Kern *et al.* 2021).

### *daf-2(gk390525)* behaves as a loss-of-function allele in fed and starved larvae

**Fig. 2.**
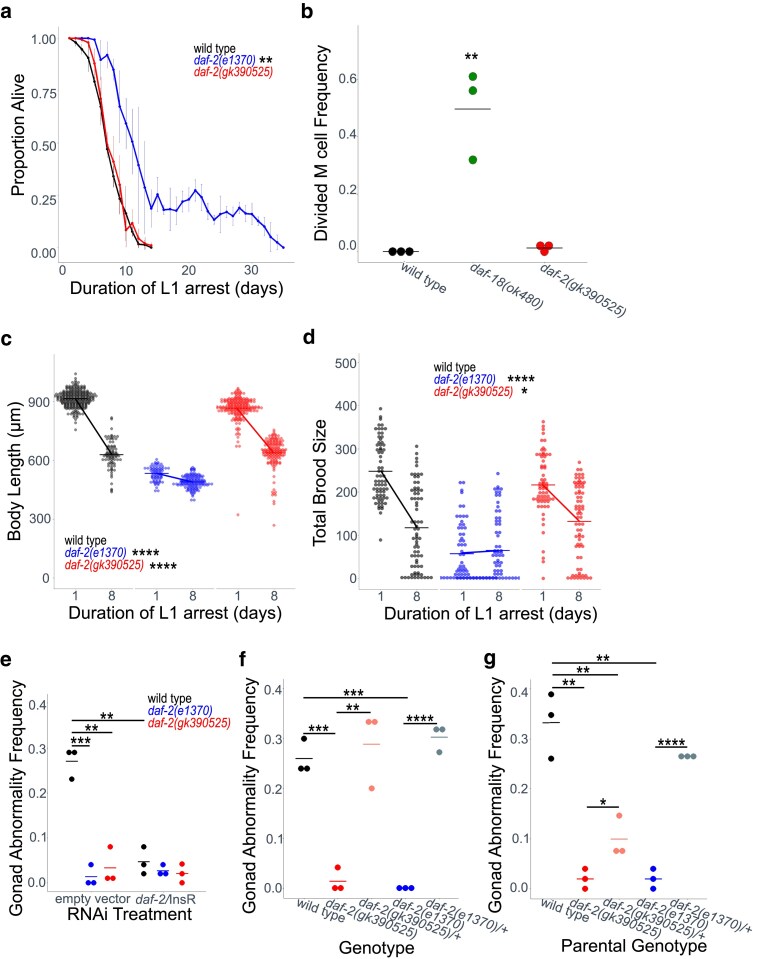
*daf-2(gk390525)* mutants display IIS loss-of-function larval phenotypes related to L1 starvation resistance. a) L1 starvation survival was scored daily for 3 biological replicates. ∼100 animals were scored for each strain in each biological replicate (median = 92, range = 15–197). The mean of replicates is plotted for each strain and day along with the SD. Two-tailed, unpaired t-tests were used to compare half-lives (median survival = 7.2 days) between wild type and each mutant (see Materials and Methods). See Supplementary Fig. 2 for individual replicates. See Supplementary Table 2 for complete data. b) M cell divisions were scored after 3 days of L1 arrest in the *daf-18­(ok480)* mutant background, and after 8 days for all other genotypes using an *hlh-8* reporter gene (Harfe *et al.* 1998) as an M cell marker in 3 biological replicates. Horizontal lines represent the mean proportion of animals with at least 1 M cell division across replicates, and points represent the proportion per replicate (scoring 100 animals) A one-sided t-test was used to assess significance. c) Worms were imaged and body length was measured after 48 h of recovery from 1 (control—arrested L1s plated 24 h after hypochlorite treatment) or 8 d L1 arrest in 3 biological replicates. Individual points represent an observation for a single animal (wild type control—*n* = 239, wild type starved—*n* = 81, *daf-2(e1370)* control—*n* = 71, *daf-2(e1370)* starved—*n* = 129, *daf-2(gk390525)* control—*n* = 201, *daf-2(gk390525)* starved—*n* = 149). d) Total brood size was scored for ∼18 individual worms in each of 3 biological replicates (median = 18, range = 11–18). Worms recovered from 8 d L1 arrest are compared with controls (24-h L1 arrest). Individual points represent individual animals. c, d) A 2-factor, linear, mixed-effects model was fit to the data with body length c) or total brood size d) as the response variable, interaction between starvation and genotype as the fixed effects, and replicate as the random effect. *P*-values for interaction terms are reported, assessing starvation-dependent effects of each genotype on phenotype. Horizontal lines represent the mean length per condition across replicates, and diagonal lines (reaction norm) connecting the means between conditions within the same genotype represent the effect of starvation. e–g) The frequency of starvation-induced gonad abnormalities is plotted for wild type and *daf-2* mutants cultured on empty vector or *daf-2* RNAi food e), for wild type along with homozygous and heterozygous *daf-2* mutant cross-progeny from wild-type mothers f), and for total progeny of wild type, homozygous, and *daf-2* heterozygous mutant mothers. Gonad abnormalities were scored in previously starved animals (8 days L1 arrest) in 3 biological replicates. See Supplementary Fig. 3 for images of the most common types of abnormalities (uterine masses, proximal germ cell tumors, and extruded vulvae). Individual points represent an observation in a population of ∼50 individuals in a single biological replicate (Fig. 2e: median = 50, range = 48–52; Fig. 2f: median = 50, range = 34–54; Fig. 2g median = 50, range = 25–60). Horizontal lines represent the mean abnormality frequency across replicates within the same condition. Homogeneity of variance across conditions and replicates was tested using Bartlett's test, and if variances were found to not be unequal they were pooled for statistical analysis. Two-tailed, unpaired t-tests with variance pooled were used to compare the frequency of gonad abnormalities between conditions and/or genotypes as indicated with bars and asterisks. a–f) *P < 0.05, **P < 0.01, ***P < 0.001, ****P < 0.0001.

DAF-2/INSR and IIS affect a variety of larval phenotypes, but the effect of *daf-2(gk390525)* on them is unknown. We assayed multiple phenotypes related to L1 arrest and recovery in *daf-2(gk390525)* and *daf-2(e1370)* mutants. IIS regulates survival during L1 arrest. *daf-2(e1370)* survived L1 arrest significantly longer than wild-type (Fig. 2b), as expected (Muñoz and Riddle 2003; Baugh and Sternberg 2006; Hibshman *et al.* 2017). However, *daf-2(gk390525)* did not affect starvation survival, despite displaying gain-of-function behavior in adults (Fig. 1).

*Caenorhabditis elegans* hatch with a single postembryonic mesoblast (M) cell that produces 18 descendants during the L1 stage that go on to produce body-wall muscle, sex muscle, and macrophage-like coelomocytes (Sulston and Horvitz 1977). However, in wild-type larvae hatched in the absence of food, the M cell does not divide, reflecting developmental arrest (Fig. 2a; Baugh and Sternberg 2006). In contrast, there are substantial M cell divisions in *daf-18(ok480)* mutants, as expected with increased IIS (Chen *et al.* 2022). Over-expression of agonistic insulin-like peptides during L1 starvation also drives M cell division (Chen and Baugh 2014). However, there were very few M cell divisions in *daf-2(gk390525)*, consistent with this allele not appreciably increasing IIS during L1 arrest (Fig. 2b).

Development is delayed following L1 arrest (Jobson *et al.* 2015), but reduction of IIS mitigates delay (Olmedo *et al.* 2020). We used image analysis to assay larval length after 48-h recovery from L1 arrest. *daf-2(e1370)* larvae were smaller than wild-type in control conditions (1 d L1 arrest, which is valuable for synchronization), as expected, but there was very little additional effect of 8 d of L1 arrest, reflecting substantial starvation resistance (Fig. 2c). Length of *daf-2(gk390525)* larvae was only modestly affected in control conditions, and there was a substantial effect of 8 d of L1 arrest on size, but the effect of starvation was significantly dampened compared with wild type. Notably, the slope of the reaction norm between 1 and 8 days of arrest is significantly smaller for *daf-2(gk390525)*, as illustrated by a significant interaction between genotype and duration of starvation in a 2-way ANOVA (Fig. 2c), reflecting increased starvation resistance. This result suggests that *gk390525* causes a loss of *daf-2* function in this context.

Brood size is reduced following extended L1 arrest (Jobson *et al.* 2015), but disruption of *daf-2/INSR* function mitigates decreased fecundity (Jordan *et al.* 2019). *daf-2(e1370)* brood size was substantially reduced in control conditions (1 day L1 arrest), as expected, but brood size was not affected by 8 d L1 arrest, again reflecting substantial starvation resistance (Fig. 2d). *daf-2(gk390525)* brood size was only modestly affected in control conditions, but the effect of starvation was significantly dampened compared with wild type. Notably, the slope of the reaction norm between 1 and 8 days of arrest is significantly smaller for *daf-2(gk390525)*, as illustrated by a significant interaction between genotype and duration of starvation in a 2-way ANOVA (Fig. 2d), reflecting increased starvation resistance. This result further supports the conclusion that *gk390525* causes a loss of *daf-2* function in L1 arrest and/or recovery.

Extended L1 arrest leads to the development of germline tumors and other developmental abnormalities in the adult gonad (Supplementary Fig. 1a and b), and disruption of *daf-2/INSR* suppresses formation of these starvation-induced abnormalities (Jordan *et al.* 2019, 2023; Shaul *et al.* 2023). *daf-2(e1370)* and *daf-2* RNAi during recovery from L1 starvation significantly suppressed formation of starvation-induced abnormalities (Fig. 2e), as expected. *daf-2(gk390525)* also significantly suppressed development of gonad abnormalities. This result further supports the conclusion that *daf-2(gk390525)* functions as a loss-of-function allele during L1 arrest and/or recovery.

*daf-2(gk390525)* appears to act as a loss-of-function allele during L1 arrest and/or recovery, but it is possible that it is a dominant-negative mutation. We tested for zygotic dominance by assaying the frequency of starvation-induced gonad abnormalities in *daf-2(gk390525)* and *daf-2(e1370)* heterozygotes from wild-type mothers. As expected, both homozygous mutants had significantly reduced abnormality frequency (Fig. 2f). Heterozygotes for both alleles had a similar abnormality frequency to wild type, and their penetrance was significantly higher than the respective homozygous mutants, supporting the conclusion that neither allele is zygotic dominant-negative.

Although *daf-2(gk390525)* does not appear to be a zygotic dominant-negative allele (Fig. 2f), this does not rule out the possibility of a dominant-negative maternal effect. Maternal dietary restriction and maternal disruption of *daf-2* both suppress formation of starvation-induced gonad abnormalities and increase reproductive success in progeny (Hibshman *et al.* 2016; Jordan *et al.* 2019). We tested for maternal dominant-negative effects by assaying the frequency of gonad abnormalities in progeny of heterozygous mothers. Because we assayed the entire brood (which contains 25% homozygous mutant, 50% heterozygotes, and 25% homozygous wild type), a maternal recessive effect should present as no suppression (comparable to the wild-type mother control), a complete maternal dominant effect should present as near complete suppression of abnormalities (comparable to the homozygous mutant mother control), and a zygotic recessive effect should present as relatively minor suppression. We ruled out a zygotic dominant effect (Fig. 2f), but it should present as substantial but incomplete suppression. Progeny from *daf-2(e1370)* heterozygotes had a significantly higher frequency of gonad abnormalities than progeny from homozygotes and were not significantly lower than progeny from wild type (Fig. 2g), suggesting that there is not a maternal dominant effect for this allele, as if it is entirely recessive, as expected. To the contrary, progeny from *daf-2(gk390525)* heterozygotes had an intermediate level of abnormalities, both significantly lower than progeny from wild type and significantly higher than progeny from homozygotes. Together, these results suggest that *gk390525* has incomplete maternal dominant-negative effects resulting in loss-of-function phenotypes in young larvae. It is possible that dominance could result from DAF-2/INSR dimerization, but it is unclear why this allele in particular would have this effect.

**Fig. 3.**
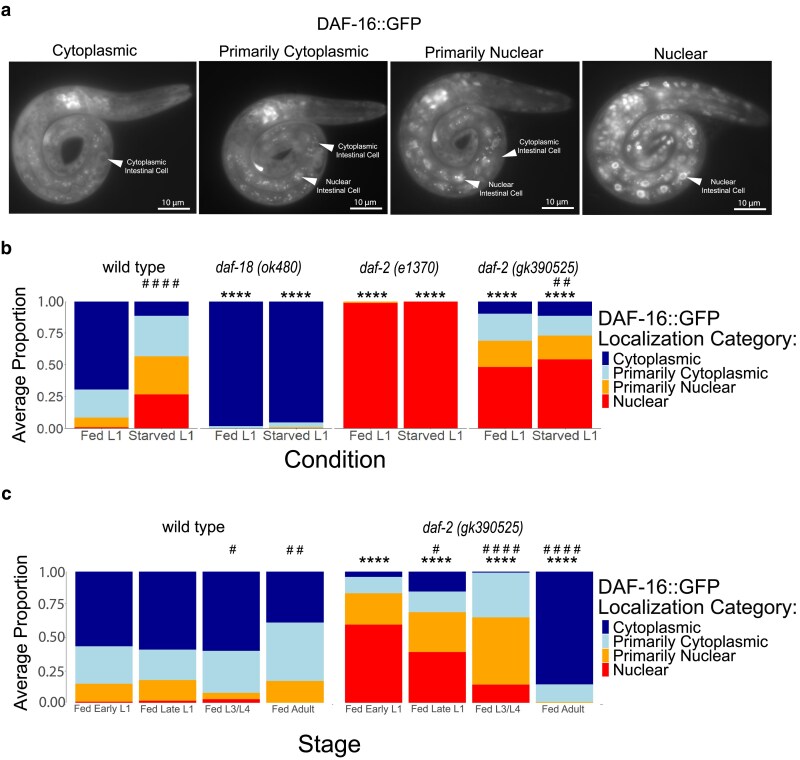
DAF-16 localization in *daf-2(gk390525)* reveals a developmental transition from loss-of-function behavior in larvae to gain-of-function behavior in adults. a) Representative images of DAF-16:GFP subcellular localization categories taken at 1000× total magnification are shown. Arrowheads and labels point out representative intestinal cells. L1 larvae were imaged during L1 arrest or shortly after hatching with food (<12 h). b) DAF-16::GFP subcellular localization was scored in ∼100 fed (18 h after hypochlorite treatment, or about 6-h L1 development) and starved (24 h after hypochlorite treatment, or about 12-h L1 arrest) L1 larvae (median = 97, range = 65–123). c) DAF-16::GFP subcellular localization was scored in ∼100 worms (median = 86, range = 54–117) throughout postembryonic development (18, 24, 48, and 72 h after hypochlorite treatment, or about 6, 12, 36, and 60 h postembryonic development, respectively). b, c) Localization was scored as nuclear, primarily nuclear, primarily cytoplasmic, or cytoplasmic in intestinal cells in 3 biological replicates. Average frequency across replicates for each category is displayed. Cochran–Mantel–Haenszel chi-squared tests were used to compare the distribution of cellular localization between genotypes and conditions. Asterisks (*) indicate significant pairwise differences between a particular mutant and wild type in the same condition. Pound signs (#) indicate significant pairwise differences between conditions in the same genotype; for b), fed and starved L1 larvae are being compared, and for c), fed larvae of the indicated stage are being compared with fed larvae of the previous stage. ****P < 0.0001, #*P* < 0.05, ##*P* < 0.01, ####*P* < 0.0001.

DAF-16/FOXO is the primary transcriptional effector of IIS and is antagonized by DAF-2/INSR and AGE-1/PI3K signaling (Murphy and Hu 2013). When IIS is active (e.g. in fed larvae), DAF-16/FOXO is phosphorylated and cytoplasmic, but when IIS is reduced (e.g. in starved larvae), DAF-16/FOXO translocates to the nucleus and regulates transcription (Henderson and Johnson 2001). We assayed DAF-16::GFP subcellular localization as a proxy for IIS activity to complement phenotypic analysis (Fig. 3a). DAF-16::GFP nuclear localization was significantly increased in starved L1 larvae compared with fed L1 larvae (Fig. 3b), as expected (Weinkove *et al.* 2006). In addition, DAF-16::GFP was almost entirely cytoplasmic and almost entirely nuclear in both conditions in *daf-18(ok480)* and *daf-2(e1370)* larvae, respectively, as expected for constitutively increased and decreased IIS. DAF-16::GFP displayed a modest but significant increase in nuclear localization in *daf-2(gk390525)* starved L1 larvae compared with fed (Fig. 3b), suggesting reduced environmental responsiveness compared with wild type. Critically, DAF-16::GFP was significantly more nuclear in *daf-2(gk390525)* fed and starved L1 larvae compared with wild type in each condition, supporting the conclusion that *daf-2(gk390525)* reduces DAF-2/INSR function in L1 larvae. Notably, these results extend the phenotypic analysis of L1 arrest and recovery (Fig. 2) to show that *daf-2(gk390525)* behaves as a loss-of-function allele in starved and fed L1 larvae.

### Disruption of DAF-2/INSR ubiquitination at K1614 has different effects on IIS in larvae and adults

*daf-2(gk390525)* behaves as a gain-of-function allele in adults but a loss-of-function in L1 larvae, leading us to hypothesize that its functional impact on IIS transitions during development. To test this hypothesis, we scored DAF-16::GFP localization over the course of larval development. Fed wild-type animals displayed relatively minor fluctuations in localization throughout development, with DAF-16::GFP being mostly cytoplasmic (Fig. 3c), as expected. In contrast, DAF-16::GFP was primarily nuclear in *daf-2(gk390525)* fed early L1 larvae, as previously observed (Fig. 3b). In fed late L1 larvae, DAF-16::GFP shifted toward being more cytoplasmic compared with early L1 larvae (Fig. 3c). Likewise, a further shift toward cytoplasmic localization was observed in L3/L4 larvae, and again in adults. Critically, DAF-16::GFP was significantly more nuclear in mutant early L1, late L1, and L3/L4 larvae compared with wild-type, but it was significantly more cytoplasmic in adults. These results demonstrate that the effect of *daf-2(gk390525)* gradually shifts during development from reducing to increasing IIS, with the transition from behaving as a loss-of-function to gain-of-function allele occurring near the onset of adulthood.

### Disruption of DAF-2/INSR ubiquitination at K1614 has different effects on IIS at different temperatures

**Fig. 4.**
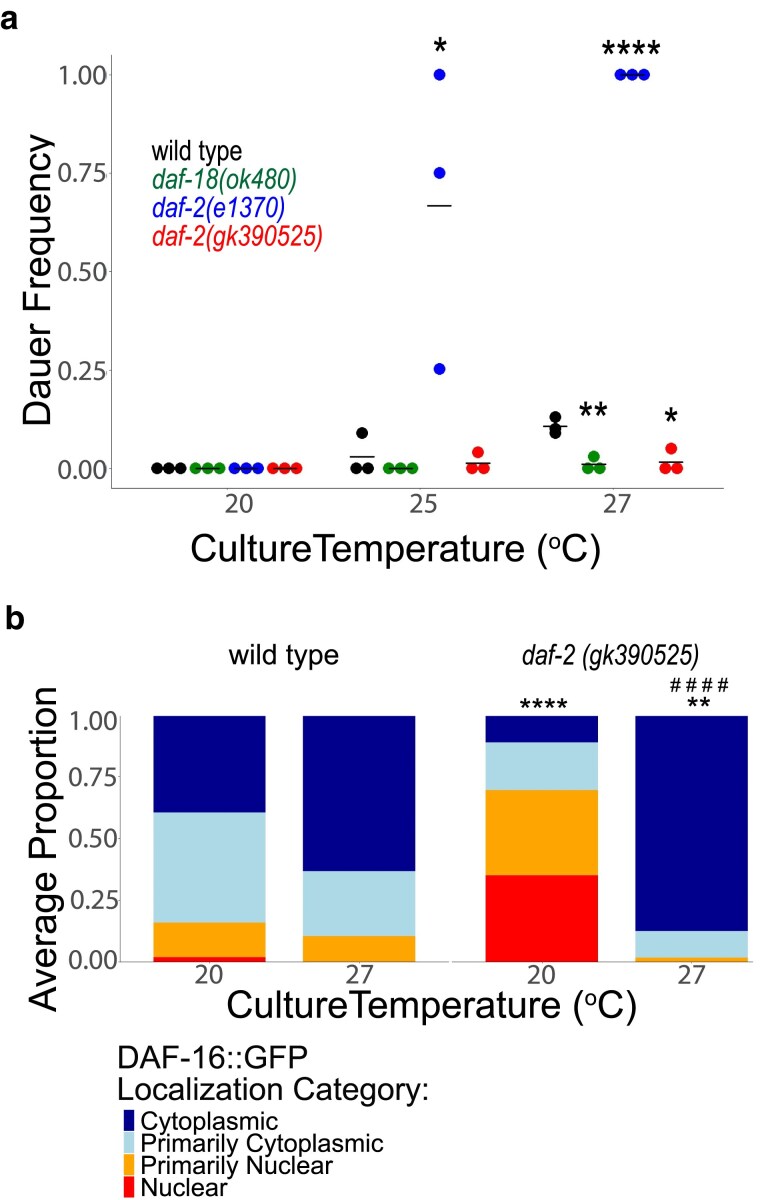
*daf-2(gk390525)* mutants display IIS gain-of-function phenotypes in larvae at elevated temperature. a) The frequency of dauer larvae was scored at 2 different temperatures in 3 biological replicates. Each individual point represents an observation for a population of ∼100 individuals in a single biological replicate (median = 100, range = 44–100). Horizontal lines represent mean dauer frequency across replicates within the same condition. Two-tailed, unpaired, pooled variance t-tests were used to compare the frequency of dauers between genotypes at each temperature. b) DAF-16 subcellular localization was scored in 3 biological replicates in ∼100 L1 larvae (median = 84, range = 63–119) grown at 20°C or 27°C. Localization was scored as nuclear, primarily nuclear, primarily cytoplasmic, or cytoplasmic in intestinal cells in 3 biological replicates (Fig. 3a). Average frequency across replicates for each category is displayed. Cochran–Mantel–Haenszel chi-squared tests were used to compare the distribution of cellular localization between conditions (genotype and temperature). Asterisks (*) indicate significant pairwise differences between genotype within a single condition (temperature), and pound signs (#) indicate significant pairwise differences between conditions within a single genotype. a, b) *P < 0.05, **P < 0.01, ****P < 0.0001, ####*P* < 0.0001.

Unlike L1 arrest, dauer arrest occurs in the L3 stage in larvae that had at least some food (enough to develop into dauer larvae) but experienced adverse conditions such as high population density, limited nutrient availability, and/or high temperature (Hu 2007). Multiple signaling pathways regulate dauer formation, including IIS. In conditions of high IIS, larvae proceed through larval development into reproductive adults, but with low IIS they arrest as dauer larvae. Given the ecological significance of dauer development and the fact that dauer formation is one of the most profound consequences of reduced IIS, we wondered how *daf-2(gk390525)* affects dauer formation. The optimal temperature range for *C. elegans* development is 15°C to 25°C, and wild-type larvae can develop as dauers at 27°C. We did not observe dauer formation in wild type, *daf-18(ok480)*, *daf-2(e1370)*, or *daf-2(gk390525)* at 20°C (Fig. 4a), as expected. However, at 25°C *daf-2(e1370)* displayed significant dauer formation, as expected, though *daf-2(gk390525)* did not. At 27°C, wild type displayed modest but reproducible dauer formation and *daf-2(e1370)* formed 100% dauers, as expected. However, *daf-18(ok480)* did not form dauers at 27°C, consistent with increased IIS, as expected. Likewise, dauer formation was significantly suppressed at 27°C in *daf-2(gk390525)* compared with wild type, suggesting increased IIS. These results suggest that *daf-2(gk390525)* behaves like a gain-of-function allele in larvae at 27°C, despite its loss-of-function behavior in larvae at 20°C.

The decision to develop as a dauer larva is made largely based on assessment of environmental conditions in the L1 stage (Schaedel *et al.* 2012). To determine whether the effects of *daf-2(gk390525)* on temperature-dependent dauer formation reflect temperature-dependent effects on IIS, we scored DAF-16::GFP localization in early L1 larvae cultured at 20°C or 27°C. We did not observe a significant difference in localization in wild-type animals (Fig. 4b), but we did observe a significant difference in *daf-2(gk390525)*, with DAF-16::GFP being predominantly nuclear at 20°C, as seen before (Fig. 3b), and predominantly cytoplasmic at 27°C. Critically, the effects of *daf-2(gk390525)* compared with wild type were significant at each temperature, with increased and decreased nuclear localization at 20°C and 27°C, respectively. These results support the conclusion that whether *daf-2(gk390525)* behaves as a loss- or gain-of-function allele in larvae depends on temperature.

### *chn-1/CHIP* antagonizes IIS during L1 arrest

**Fig. 5.**
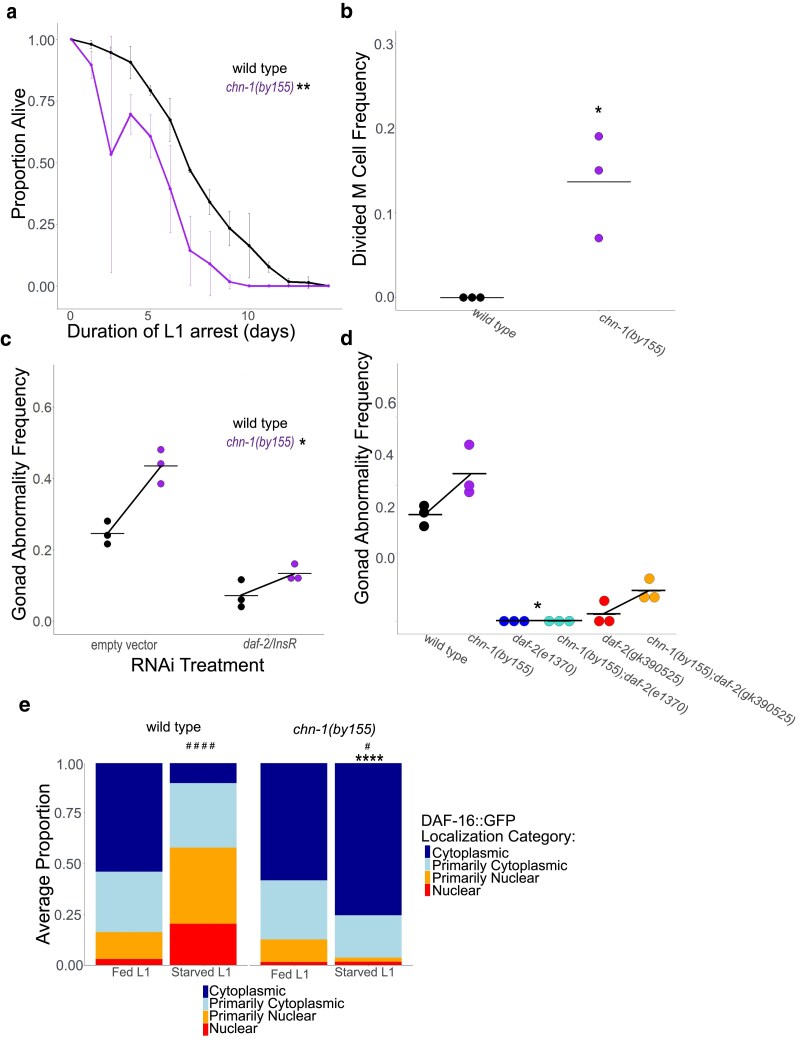
*chn-1/CHIP* antagonizes IIS during L1 arrest. a) L1 starvation survival was scored daily for 3 biological replicates. Approximately 100 animals were scored for each strain in each biological replicate (median = 97, range = 36–152). The mean of replicates is plotted for each strain and day along with the SD. Two-tailed, unpaired variance t-tests were used to compare half-lives between wild type and each mutant (see Materials and Methods). See Supplementary Fig. 4 for individual replicates. See Supplementary Table 2 for complete data. b) M cell divisions were scored after 8 days of L1 starvation using an *hlh-8* reporter gene (Harfe *et al.* 1998) as an M cell marker in 3 biological replicates. Horizontal lines represent the mean proportion of animals with at least 1 M cell division across replicates, and points represent the proportion per replicate (scoring 100 animals). A one-sided t-test was used to assess significance. See Supplementary Table 2 for complete survival data. c and d) The frequency of adults with starvation-induced gonad abnormalities was scored at the onset of egg laying in worms that were starved for 8 d as L1 larvae. Three biological replicates were included. A 2-way ANOVA was used to analyze epistasis between each pair of perturbations, and the *P*-value for a nonlinear interaction (epistasis) between the 2 perturbations is reported. Individual points represent abnormality frequency in a population of ∼50 individuals (Fig. 5c: median = 50, range = 50–52; Fig. 5d: median = 50, range = 26–50) as a single biological replicate. Horizontal lines represent the mean abnormality frequency across replicates within the same condition, and diagonal lines (reaction norms) connecting the means represent the effect of perturbations through RNAi or mutation. c) Wild type or *chn-1* mutants were recovered from L1 arrest on empty vector or *daf-2* RNAi food. Two-way ANOVA P interaction = 0.017. d) Worms of the indicated genotype were recovered and gonad abnormalities were scored. Two-way ANOVA *P* interaction for *chn-1(by55)* × *daf-2(e1370)* = 0.034; 2-way ANOVA *P* interaction for *chn-1(by55)×daf-2(gk390525)* = 0.38. a–d) *P < 0.05, **P < 0.01. e) DAF-16::GFP subcellular localization was scored in ∼100 fed (18 h after hypochlorite treatment, or about 6-h L1 development) and starved (24 h after hypochlorite treatment, or about 12-h L1 arrest) L1 larvae (median = 105, range = 72–138). Localization was scored as nuclear, primarily nuclear, primarily cytoplasmic, or cytoplasmic in intestinal cells in 3 biological replicates (Fig. 3a). Average frequency across replicates for each category is displayed. Cochran–Mantel–Haenszel chi-squared tests were used to compare the distribution of cellular localization between conditions (genotype and fed/starved). Asterisks (*) indicate significant pairwise differences between the mutant and wild type within the same condition. Pound signs (#) indicate significant pairwise differences between conditions (fed vs starved) within a single genotype. *P < 0.05, #*P* < 0.05, ####*P* < 0.0001.

The opposite effects of *daf-2(gk390525)* on IIS in larvae and adults suggest that DAF-2/INSR K1614 is not ubiquitinated in L1-stage larvae. However, the protein is potentially ubiquitinated at other residues (Tawo *et al.* 2017), raising the question of whether CHN-1/CHIP regulates IIS in larvae as it does in adults. We interrogated the function of *chn-1/CHIP* during L1 arrest to address this question. Although *daf-2(gk390525)* did not affect starvation survival during L1 arrest (Fig. 2a), *chn-1(by155)* significantly decreased survival (Fig. 5a). In addition, although *daf-2(gk390525)* had little effect on M cell divisions during L1 arrest (Fig. 2b), *chn-1(by155)* displayed an arrest-defective phenotype with a significant proportion of animals having M cell divisions (Fig. 5b). These results suggest that *chn-1/CHIP* negatively regulates DAF-2/INSR activity during L1 arrest.

We extended our phenotypic analysis of *chn-1/CHIP* to include starvation-induced gonad abnormalities, and we used epistasis analysis to determine whether the effects of *chn-1/CHIP* depend on *daf-2/INSR*. Mutation of *chn-1/CHIP* significantly increased the frequency of starvation-induced abnormalities (Fig. 5c), suggesting that CHN-1/CHIP negatively regulates IIS in larvae, though it is unclear from this if it functions during L1 arrest, recovery, or both. *daf-2/INSR* RNAi during recovery partially suppressed abnormalities in the *chn-1(by155)* background, and there was a significant interaction (nonadditivity) between *chn-1(by155)* and *daf-2/INSR* RNAi based on a 2-way ANOVA (Fig. 5c). This interaction, which can be visualized as the difference in empty vector and *daf-2* RNAi slopes for the reaction norms between wild type and *chn-1(by155)*, suggests epistasis. *daf-2(e1370)* completely suppressed abnormalities in the *chn-1(by155)* background (Fig. 5d), revealing complete epistasis. As a central E3 ligase, it is likely that CHN-1/CHIP has multiple protein targets, but the fact that *daf-2/INSR* is epistatic to *chn-1/CHIP* suggests that this *chn-1(by155)* phenotype results from lack of ubiquitination of DAF-2/INSR. However, *daf-2(gk390525)* only partially suppressed abnormalities in the *chn-1(by155)* background, and there was not a significant interaction between the 2 mutations based on 2-way ANOVA (Fig. 5d; see legend), suggesting additive function, or lack of epistasis. In summary, relatively strong loss of *daf-2/INSR* function, resulting from RNAi or the *e1370* allele, resulted in epistasis between *daf-2/INSR* and *chn-1/CHIP*, consistent with CHN-1/CHIP targeting DAF-2/INSR in larvae. However, epistasis was not observed with *daf-2(gk390525)*, suggesting that the K1614E substitution interferes with ubiquitination in this context.

We complemented phenotypic analysis of *chn-1/CHIP* by assaying DAF-16::GFP subcellular localization. Mutation of *chn-1/CHIP* did not affect DAF-16::GFP localization in fed L1 larvae (Fig. 5e). However, mutation of *chn-1/CHIP* significantly increased cytoplasmic localization in starved L1 larvae, suggesting that CHN-1/CHIP provides conditional, nutrient-dependent regulation of IIS in L1-stage larvae. In conclusion, our results suggest that CHN-1/CHIP antagonizes IIS during L1 arrest, as in adults, but not in fed L1 larvae. However, in contrast to adults, they also suggest that an amino acid other than K1614 is the functional site of DAF-2/INSR ubiquitination during L1 arrest.

## Conclusions

This work reveals developmental and conditional regulation of IIS activity via ubiquitination of DAF-2/INSR. We show that *daf-2(gk390525)*, which has one of several CHN-1/CHIP-dependent DAF-2 ubiquitination sites mutated, acts as a gain-of-function allele in adults and larvae cultured at elevated temperature, but that it acts as a loss-of-function allele in fed and starved larvae cultured at 20°C with a maternal dominant-negative effect. In contrast, *chn-1(by155)* increases IIS activity during L1 arrest, as in adults, but not in fed L1 larvae. Furthermore, epistasis analysis suggests CHN-1/CHIP targets DAF-2/INSR during L1 arrest, suggesting that it ubiquitinates an amino acid not disrupted by *daf-2(gk390525)*, though it is possible that CHN-1/CHIP targets other proteins for ubiquitination in this context. Overall, our results suggest that CHN-1/CHIP ubiquitinates DAF-2/INSR, enforcing negative regulation of IIS, in starved L1 larvae, larvae cultured at elevated temperature (27°C), and in adults. However, we found no evidence that CHN-1/CHIP ubiquitinates DAF-2/INSR in fed larvae at 20°C, though it is possible that a different ubiquitin ligase targets DAF-2/INSR in this context. *daf-2(gk390525)* increases IIS activity in adults because disruption of ubiquitination leads to elevated DAF-2/INSR protein levels (Tawo *et al.* 2017), but it is unclear why this allele reduces IIS activity in larvae. We speculate that the mutation disrupts DAF-2/INSR function independent of its effect on ubiquitination, such that the net effect is increased function in contexts where K1614 is subject to ubiquitination but decreased function in contexts where it is not. Notably, the lysine-to-glutamic acid change in *daf-2(gk390525)* (K1614E) could have significant effects on DAF-2/INSR structure and function given substitution of a positively charged amino acid with a negatively charged one. Though biochemical and phenotypic evidence suggests that the DAF-2 K1614 residue acts as a site for mono-ubiquitination leading to degradation (Tawo *et al.* 2017), we cannot rule out other functional roles for ubiquitination at this site. Researchers should interpret phenotypes resulting from *daf-2(gk390525)* carefully since it has complex, context-dependent effects on IIS. In summary, CHN-1/CHIP-dependent ubiquitination of DAF-2/INSR is not unitary but instead modifies IIS in different ways depending on conditions.

## Supplementary Material

jkaf009_Supplementary_Data

## Data Availability

The authors affirm that all data necessary for confirming the conclusions of the article are present within the article, figures, and tables. Supplementary Tables 1 and 2 contain replicate-level data for the lifespan and starvation survival analyses in Figs. 1a, 2a, and 5a. Strains are available upon request. Supplemental material available at G3 online.
