# Supplementary material for: Developmental and conditional regulation of DAF-2/INSR ubiquitination in Caenorhabditis elegans
Source: G3 (Bethesda). 2025 Jan 22;15(3):jkaf009. doi: 10.1093/g3journal/jkaf009 (PMC11917487; doi:10.1093/g3journal/jkaf009)
Supplement: jkaf009_Supplementary_Data [file jkaf009_supplementary_data.zip › Supplemental_Material_Legends_G3-2025-405641.docx]

**SUPPLEMENTAL MATERIAL LEGENDS**

Figure S1. Independent biological replicates show that *daf-2(gk390525)* reproducibly reduces adult lifespan (related to Fig. 1A). (A-C) Adult lifespan was scored daily for three biological replicates (A, B, and C) with 50 worms per replicate. See Table S1 for complete results. ** < 0.01, **** P < 0.0001; log-rank test on individual replicates.

Figure S2. Independent biological replicates suggest that *daf-2(gk390525)* does not have an appreciable effect on starvation survival (related to Fig. 2A). (A-C) L1 starvation survival was scored daily in three replicates (A, B, and C) with ~100 animals for each strain in each replicate (median = 92, range = 15-197). A logistic regression was used to fit a curve to each strain in each replicate, which is included in the plots along with individual data points (proportion alive in each strain in each replicate at each time point). Unpaired t-tests were used to compare half-lives inferred from regression (median survival) between wild type and each mutant (see Materials and Methods). See Table S2 for complete data.

Figure S3. Extended L1 arrest followed by recovery with *E. coli* HT115 causes developmental abnormalities of the gonad on the first day of egg laying (related to Fig. 2E-G and 5D; Jordan *et al.* 2019). A) Representative image of a healthy adult without obvious gonad abnormalities after expended L1 arrest. B) Representative image of an adult with the most common gonad abnormalities observed after extended L1 arrest, including a differentiated uterine mass, a proximal germ cell tumor, and an extruded vulva. A,B) Images of wild-type adults following extended L1 arrest (8 days) were taken at 400x total magnification 72 hr after plating with food at 20°C. Images were taken with differential interference contrast (DIC). FIJI and Adobe Illustrator were used for adjusting brightness/contrast and stitching images. Relevant tissues, organs, and abnormalities are outlined or indicated with arrowheads and labels.

Figure S4. Independent biological replicates show that *chn-1(by155)* reproducibly decreases starvation survival (related to Fig. 5A). A-C) L1 starvation survival was scored daily in three replicates (A, B, and C) with ~100 animals for each strain in each replicate (median = 97, range = 36-152). A logistic regression was used to fit a curve to each strain in each replicate, which is included in the plots along with individual data points (proportion alive in each strain in each replicate at each time point). Unpaired t-tests were used to compare half-lives inferred from regression (median survival) between wild type and each mutant (see Materials and Methods). See Table S2 for complete data.

Table S1. Summary statistics and complete data for lifespan assay.

Table S2. Summary statistics and complete data for starvation survival assay.
