# Supplementary figures and images for: Developmental and conditional regulation of DAF-2/INSR ubiquitination in Caenorhabditis elegans
Source: G3 (Bethesda). 2025 Jan 22;15(3):jkaf009. doi: 10.1093/g3journal/jkaf009 (PMC11917487; doi:10.1093/g3journal/jkaf009)

# Figure S1

(a)

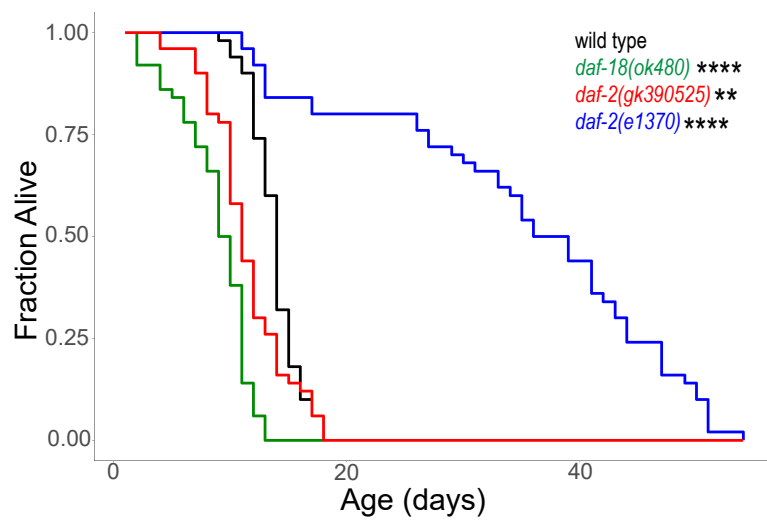

(b)

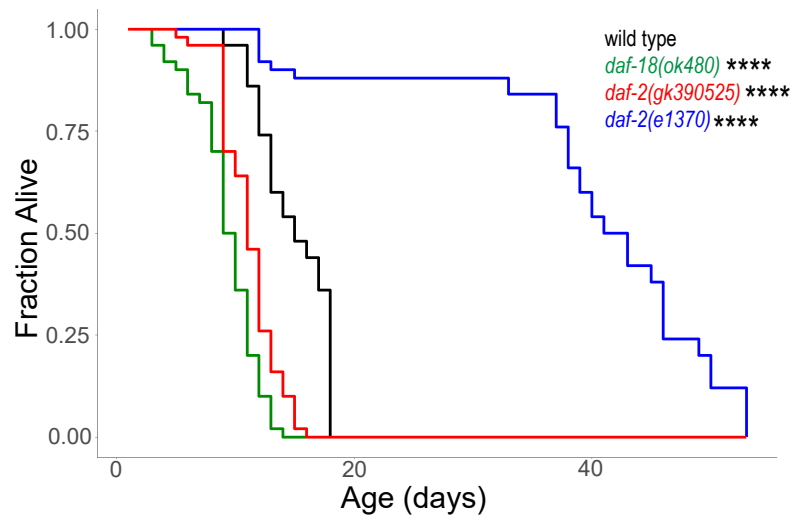

(c)

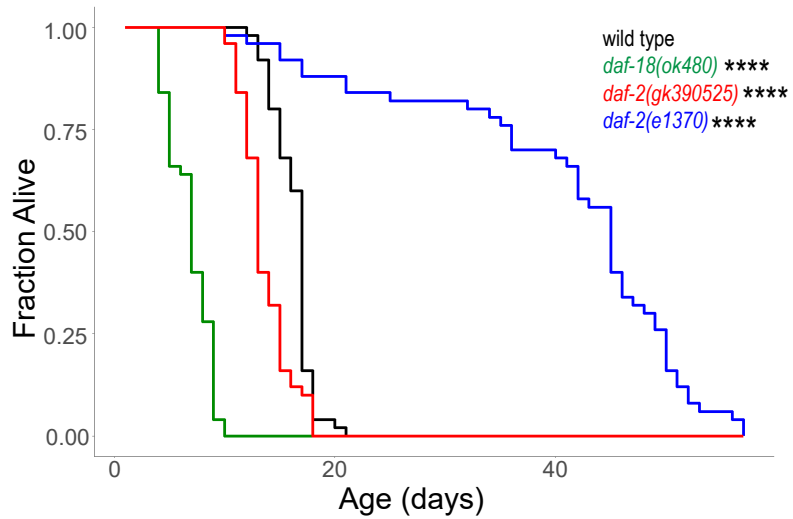

Supplement: jkaf009_Supplementary_Data [file jkaf009_supplementary_data.zip › Figure_S1_G3-2025-405641.pdf]

# Figure S2

(a)

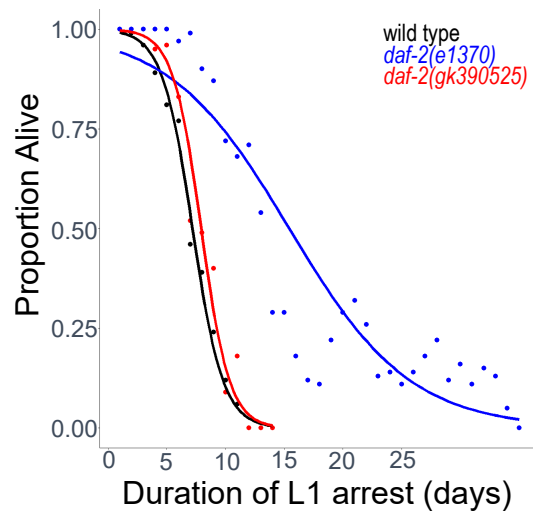

(b)

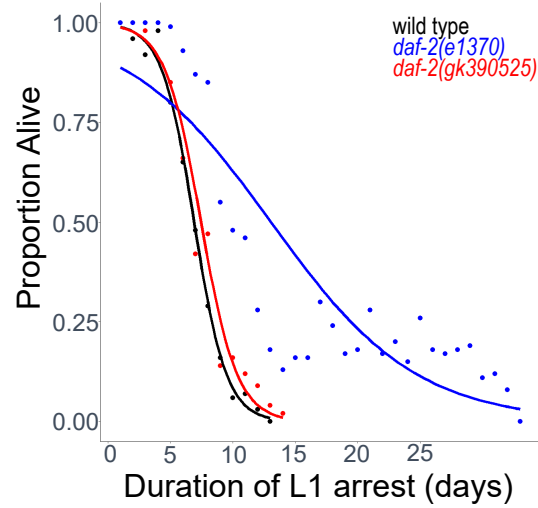

(c)

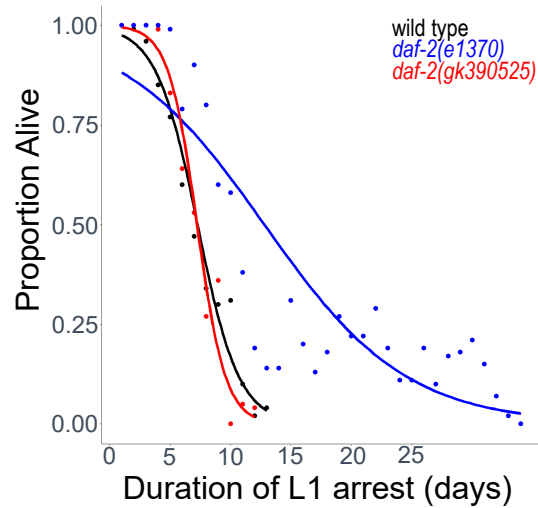

Supplement: jkaf009_Supplementary_Data [file jkaf009_supplementary_data.zip › Figure_S2_G3-2025-405641.pdf]

# Figure S3

(a)

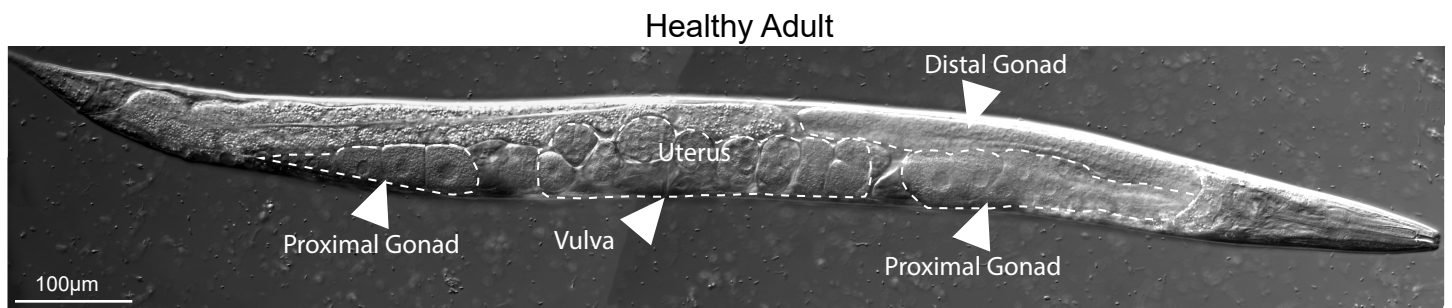

(b)

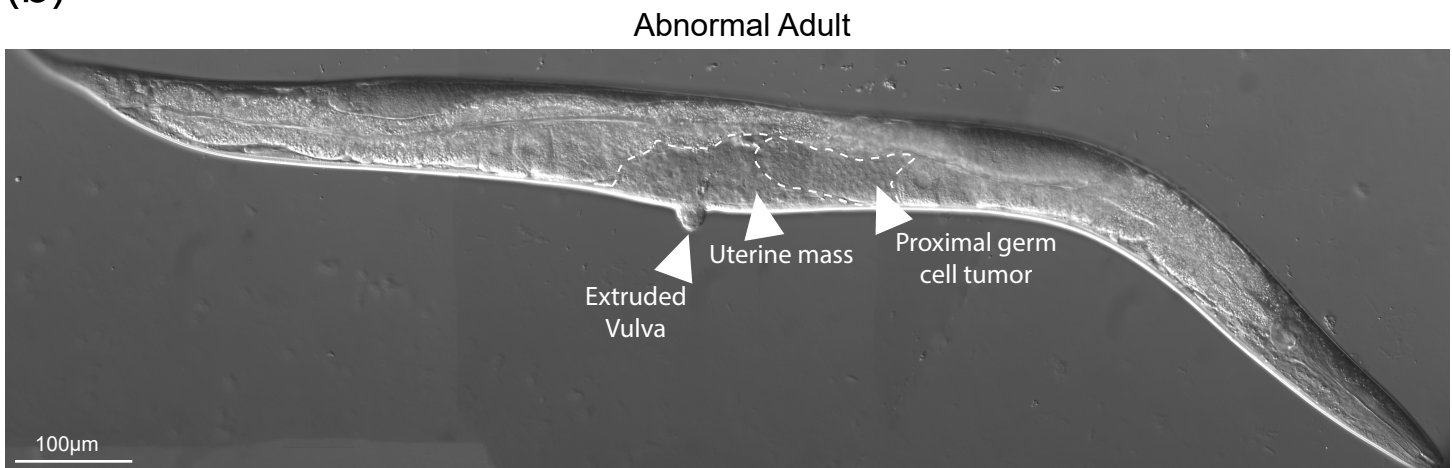

Supplement: jkaf009_Supplementary_Data [file jkaf009_supplementary_data.zip › Figure_S3_G3-2025-405641.pdf]

# Figure S4

(a)

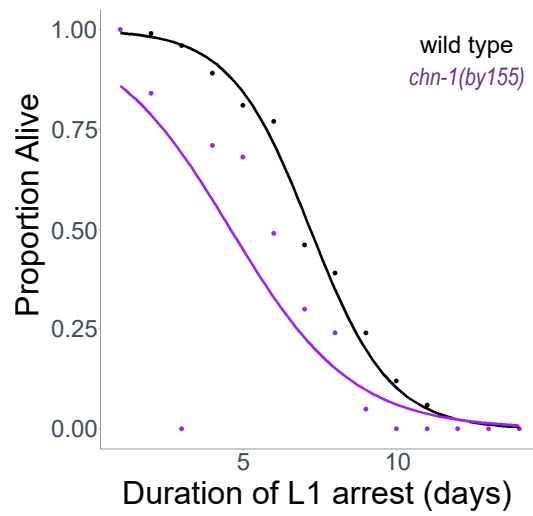

(b)

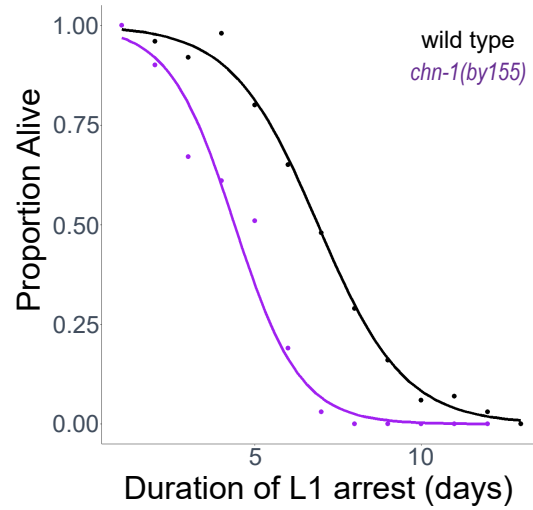

(c)

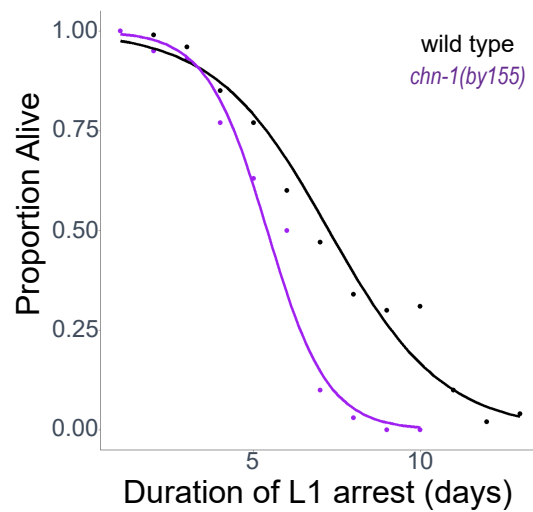

Supplement: jkaf009_Supplementary_Data [file jkaf009_supplementary_data.zip › Figure_S4_G3-2025-405641.pdf]
